# Statistical Analysis of Factors Associated with Diarrhea in Yemeni Children under Five: Insights from the 2022–2023 Multiple Indicator Cluster Survey

**DOI:** 10.1007/s44197-024-00253-1

**Published:** 2024-06-10

**Authors:** Ali Satty, Mohyaldein Salih, Faroug A. Abdalla, Ashraf F. A. Mahmoud, Elzain A. E. Gumma, Gamal Saad Mohamed Khamis, Ahmed M. A. Adam, Abaker A. Hassaballa, Omer M. A. Hamed, Zakariya M. S. Mohammed

**Affiliations:** 1 Department of Mathematics, College of Science, Northern Border University, Arar, Saudi Arabia; 2 Department of Computer Science, College of Science, Northern Border University, Arar, Saudi Arabia; 3 Department of Finance, College of Business Administration, Northern Border University, Arar, Saudi Arabia

**Keywords:** Childhood Diarrhea, Logistic Regression, Household Characteristics, Multiple indicator Cluster Survey

## Abstract

Diarrheal disease remains a significant cause of preventable morbidity and mortality in the pediatric population, particularly among children below five years of age. Although the occurrence of diarrheal episodes is on the decline, its impact continues to escalate at a concerning rate among children under the age of five, especially in developing countries. The objective of this paper is to investigate the factors associated with diarrhea in Yemeni children younger than five years, drawing on data from the latest edition of the Multiple Indicator Cluster Survey (MICS) Yemen conducted in 2022–2023. To identify factors associated with the prevalence of childhood diarrhea, bivariate analysis and multivariable logistic regression were utilized. The findings of this study suggest that age group 6–23, unimproved sanitation, and low-income households are associated with high risk of diarrhea in children under five years of age in Yemen. The study contributes additional evidence regarding factors that should be prioritized in public health strategies geared towards reducing diarrheal prevalence among Yemeni children.

## Introduction

Diarrheal disease constitutes a significant public health challenge in lower-to middle-income countries, resulting in a high mortality rate among children below the age of five. Despite being preventable, diarrhea remains the second-highest cause of mortality among children under five. Annual reports indicate approximately 1.7 billion incidences of childhood diarrheal diseases, culminating in the demise of an estimated 525,000 children under five, which represents 8% of global deaths [1]. There has been a global decline in the incidence of diarrhea in children over the past two decades, and its prevalence remains very high in developing countries, including the Eastern Mediterranean countries. In such places, children under the age of five are constantly at risk of death from diarrhea [1, 2].

WHO reports that infectious diarrheal diseases are prevalent among developing countries [3]. In the Eastern Mediterranean Region, as delineated by WHO, a cumulative total of 267,052 cases of acute watery diarrhea were documented between January 1 and August 31, 2023, from eight member countries; notably, Yemen reported 3,878 of these cases [4]. In Yemen, the prevalence of watery diarrhea among children under the age of five reaches 24% across Eastern Mediterranean countries [4]. The ongoing civil war in Yemen since early 2015 has precipitated a dire humanitarian crisis [5], and given Yemen’s economic status relative to its neighbors, this has further debilitated an already struggling health system. Therefore, the impact on pediatric health is profound. Despite the known significance of diarrhea as a cause for morbidity and mortality among children under five in developing countries like Yemen, risk factors for childhood diarrhea remain insufficiently characterized [6, 7]. This highlights an urgent requirement for comprehensive research aimed at elucidating causative elements for childhood diarrhea in children under the age of five. Moreover, there is a critical need to enhance surveillance and laboratory capacities in Yemen to address this public health concern effectively.

The literature on pediatric diarrhea in Yemen, particularly among children under the age of five, is sparse, with limited regional studies such as the investigation into the management of diarrhea in Aden Governorate by Abdulla et al. [7], and an assessment of risk factors in Taiz Governorate conducted by Al-Badani et al. [8]. Another pertinent study aimed at enhancing the understanding of common pediatric diseases including diarrhea within Shuhair Governorate was articulated in [9]. Additionally, research conducted by Bahartha and AlEzzi [10] explored the risk factors associated with diarrhea in children younger than five years old in Mukalla Governorate. These studies primarily focus on the implications of childhood diarrhea within specific Governorates rather than addressing the problem across the entire country. Consequently, this study is motivated by the need to conduct a comprehensive analysis of diarrheal factors affecting children under five years old across all Yemeni Governorates. The main objective of this paper is to investigate the factors associated with diarrhea among Yemeni children aged less than five years by utilizing data from the most recent Multiple Indicator Cluster Survey (MICS) 2022–2023. The importance of this study stems from the use of its results to help policy makers and practitioners in the field of public health to prevent diarrheal infections in Yemeni children under the age of five.

## Methods

### Study Design

This study is a retrospective cross-sectional study, utilizing secondary data gathered from the Multiple Indicator Cluster Survey (MICS) conducted in Yemen during the 2022–2023 period. The study population was consisted of children from Yemen who were below the age of five years (ranging from 0 to 59 months), including all living children participated in the survey.

### Description of Multiple Indicator Cluster Survey (MICS)

MICSs are a series of internationally conducted household surveys that have garnered support from the United Nations Children’s Fund (UNICEF) [11]. These surveys provide robust and reliable data concerning women and children [12]. The MICS utilizes a cross-sectional study framework, implementing an extensive two-stage sampling process. This process begins with the selection of census enumeration areas within each designated stratum, with selections made in proportion to the number of households contained within each enumeration area. Subsequently, households within each enumeration area are chosen systematically to create survey clusters. A comprehensive description of MICS sampling procedures can be found in [11], while the MICS program’s website allows for free access to datasets upon request.

### Data Collection

The dataset acquired from MICS was used, including a sample of 19,651 children aged below five years. As the study investigates the association between childhood diarrhea and socio-demographic and household factors, the following variables are included in the analysis. The outcome variable of interest is diarrheal illnesses among children, specifically those under the age of five. Diarrhea in a child is defined as the occurrence of three or more loose or liquid stool passages per day, or the presence of blood in stools as reported by the child’s mother or primary caregiver within the fortnight preceding the survey administration. For the purposes of this research, “mother” encompasses both biological mothers and female guardians who are the primary caregivers and cohabit with the child. While the exploratory variables under consideration encompass the age of the child, stratified into four groups 0 to 5 months, 6 to 11 months, 12 to 23 months, and 24 to 59 months; the sex of the child (male or female); and the educational level attained by the mother, categorized as no formal education, primary education, secondary education, and tertiary education (college or advanced degree); administrative division of residence; economic status of the household as indicated by the wealth quintile (poorest, poor, middle, rich, richest); urbanity of domicile (rural or urban habitats); quality of drinking water sources (sanctioned as safe or not safe); and status of sanitation facilities (improved, unimproved). In this study, we categorized the variables pertaining to the sources of drinking water, based on the categorization delineated in MICS report [11]. Unimproved drinking water sources encompass household drinking water obtained from unprotected wells, springs without safeguarding, surface water bodies, and other similar sources. Conversely, improved water sources comprise a range of options such as piped water into dwellings, wells with protective measures, springs with contamination protection, systems for collecting rainwater, water distributed by tanker trucks, and packaged water including bottled and sachet varieties, as well as carts equipped with small tanks. Finally, households were classified as having unimproved sanitation facilities if they relied upon pit latrines without a constructed slab or platform, suspended latrines, or bucket-based latrines. Conversely, improved sanitation was characterized by the presence of flush or pour-flush toilets, pit latrines equipped with a slab, ventilated improved pit latrines, or composting toilets.

### Data Analysis

Initial descriptive statistical analyses are conducted to summarize the dataset concerning the prevalence of diarrhea in children younger than five years of age, with a particular emphasis on examining socio-demographic and household determinants. Frequencies and percentages were calculated to describe the distribution of diarrhea cases within each category of the explanatory variables, offering insights into the prevalence rates across different demographic groups. Subsequently, a chi-square test was conducted to assess the bivariate association between diarrhea occurrence and the various factors. This test allowed for the identification of statistically significant association between the presence of diarrhea and sociodemographic or household characteristics. Since the dataset used in this study is based on a complex survey design that involves probabilistic, stratified, and multistage sampling with varying weights for observations, the survey logistic regression analysis is utilized to account for the complexities in sampling design to facilitate valid inferences [13]. Simple logistic regression model was employed to further elucidate the impact of individual determinant on the likelihood of diarrhea occurrence. By estimating the unadjusted prevalence odds ratios (UOR) and associated 95% confidence intervals, this analysis revealed the strength and direction of the association between each determinant and the likelihood of diarrhea. Finally, multivariable logistic regression model is used to simultaneously assess the effects of multiple predictors of diarrhea. This simultaneous modeling allows the calculation of adjusted prevalence odds ratios (AOR) as a measure of contribution of individual determinant after adjusting for other factors included in the model. This comprehensive approach enabled a nuanced understanding of the factors of diarrhea in children under five, shedding light on the relative contributions of sociodemographic and household characteristics to the risk of this prevalent childhood illness. Data analysis was facilitated using IBM SPSS Version 26.

## Results

### Descriptive Statistics and Prevalence

Table 1 summarizes the characteristics of children under five included in the study, the prevalence estimates across exploratory variables, and the association between diarrhea prevalence and these variables. The study included 19,561 children of whom 51.5% are males, 72.4% of the children lived in rural areas, 79.2% used improved drinking water, 60% had improved sanitation, and 40.4% of mother or caregivers had no formal education. Age distribution was: 0–5 months (9.8%), 6–11 months (10.5%), 12–23 months (20.1%), 24–35 months (21.1%), 36–47 months (20.5%), and 48–59 months (20.5%). Wealth status ranged from 17.2% in the richest category to 23.7% in the poorest.

**Fig. 1:**
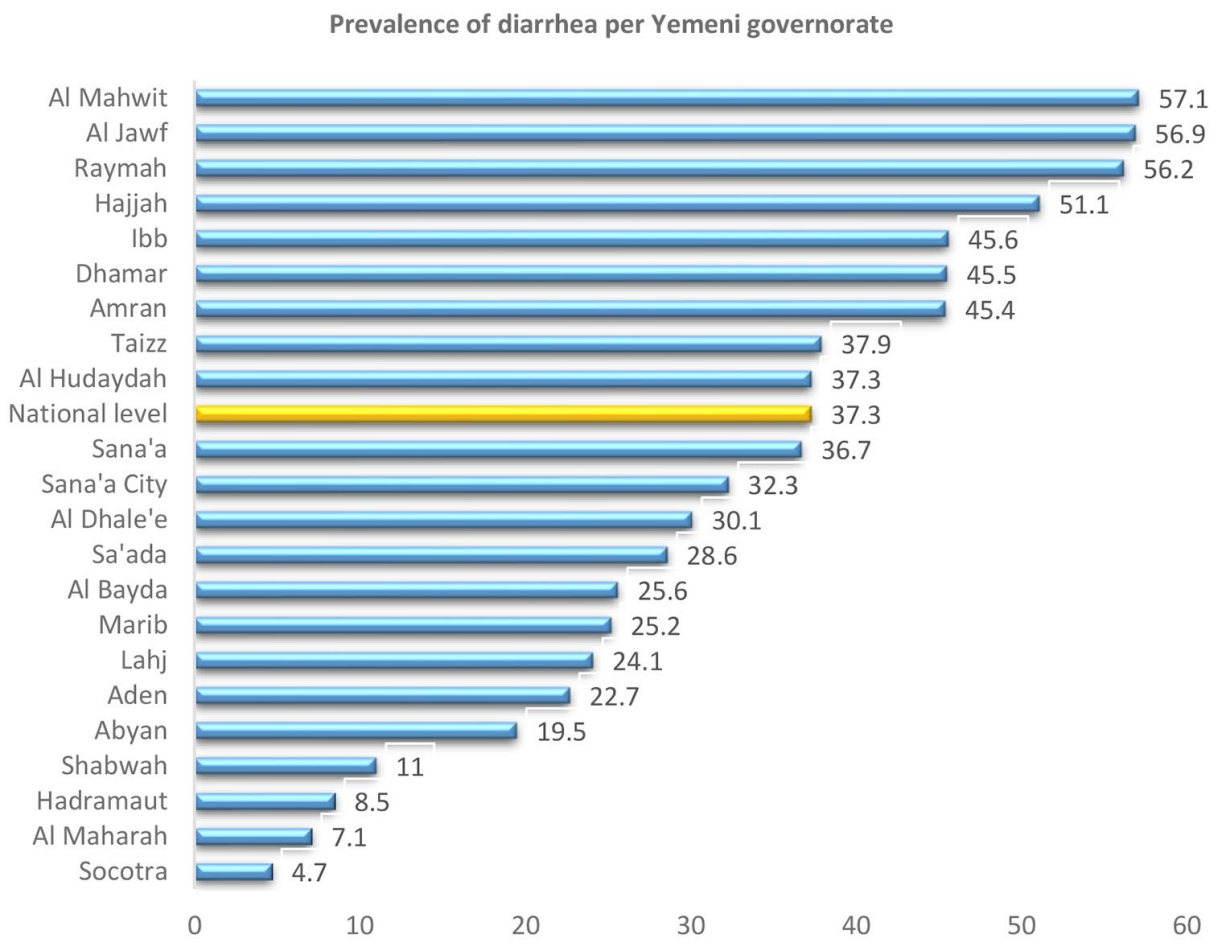
Prevalence of diarrhea per Yemeni governorate

The overall prevalence of childhood diarrhea was 37.3. Statistically significant variation in diarrhea prevalence according to exploratory variables were found. The prevalence was higher in rural areas (39.3% vs. 32.6% in urban areas), poorest households (44.7%), children aged 6–11 months (47.4%) and 12–23 months (48.7%), males (38.7%), households with unimproved water sources (43.2%), and households without improved sanitation (43.1%). Figure 1 shows diarrhea prevalence across Yemeni governorates, with AlMahwit (57.1%) and AlJawf (56.9%) having the highest rates, and Socotra the lowest (4.7%).

### Multivariable Analysis

Table 2 reports the results of the logistic regression analysis, which encompass both unadjusted prevalence odds ratios obtained from a logistic regression with one predictor (Model I) and adjusted prevalence odds ratios obtained from multivariable logistic regression (Model II). The unadjusted logistic regression results demonstrates an inverse association between children age and the incidence of childhood diarrheal episodes. Specifically, children in the 12–23 month age category (UOR: 2.42; 95% CI: 2.199, 2.667) exhibited a higher odd of childhood diarrhea, compared to their older counterpart aged 48–59 months. This trend was similarly observed in children aged 6–11 months (UOR: 2.30; 95% CI: 2.055, 2.578). An increase in odds of childhood diarrhea by more than 20% was also noted among children aged 24–35 months (UOR: 1.48; 95% CI: 1.341 to 1.629), 0–5 months (UOR: 1.28; 95% CI: 1.134 to 1.443) and 36–47 months (UOR: 1.20; 95% CI: 1.088 to 1.327). Gender disparities emerged from the analysis as well; male children faced a statistically significant increase of 12% in diarrheal odds (UOR: 1.12; 95% CI: 1.053, 1.183) compared to female children. The educational level of mothers or primary caregivers has been statistically related to childhood diarrhea. Specifically, a mother’s or caregiver’s limited education correlates with a 49% increased likelihood of diarrhea in children (UOR: 1.49; 95% CI: 1.280, 1.730), relative to their counterparts whose mothers/caregivers possess higher levels of education. Additionally, the socioeconomic standing of a household, as measured by wealth status, demonstrates a significant association with the prevalence of childhood diarrhea. Children from households classified within the lowest two wealth quintiles, poorest (UOR: 2.61; 95% CI: 2.360, 2.875) and poor (UOR: 2.42; 95% CI: 2.190, 2.682), exhibit higher odds of diarrhea compared to those from households in the richest wealth quintile. In contrast, children residing in households categorized within the middle and upper-middle wealth brackets show increased odds of suffering from diarrheal diseases by 92% (UOR: 1.92; 95% CI: 1.726–2.124) and 73% (UOR: 1.73; 95% CI: 1.557–1.917), respectively, when compared to children from the wealthiest families. The study also illuminates geographical disparities, evidencing a reduction childhood diarrhea risk among urban-dwelling children by approximately 25% (UOR: 0.75; 95% CI: 0.699–0.798) when compared to their rural counterparts. An increase in the risk of childhood diarrhea was also observed in households with unimproved drinking water sources by 36% (UOR: 1.36; 95% CI: 1.265, 1.456), compared to those with improved drinking water sources. Regarding sanitation, the results showed a 49% increase in the odd of childhood diarrhea in households with unimproved sanitation (UOR: 1.49; 95% CI: 1.405, 1.581).

**Table 1:** Prevalence of diarrhea for the socio-demographic and households characteristics

Variables	Frequency *N* %	Prevalence	*P*-value
**Child age (months)**			
0–5	1909 (9.8)	33.4	$$ <0.001$$
6–11	2061 (10.5)	47.4	
12–23	3927 (20.1)	48.7	
24–35	4119 (21.1)	36.7	
36–47	4013 (20.5)	32.0	
48–59	3532 (18)	28.2	
**Gender**			
Male	10,073 (51.5)	38.7	$$ <0.001$$
Female	9488 (48.5)	36.1	
**Mother’s education**			
No formal education	7909 (40.4)	38.5	$$ <0.001$$
Primary education	5002 (25.6)	38.6	
Lower secondary education	2394 (12.2)	36.5	
Upper secondary education	3360 (17.2)	36.0	
Higher education	896 (4.6)	29.6	
**Household Wealth**			
Poorest	4630 (23.7)	44.7	$$ <0.001$$
Poor	4000 (20.4)	43.0	
Middle	3750 (19.2)	37.3	
Rich	3817 (19.5)	34.9	
Richest	3364 (17.2)	23.7	
**Place of residence**			
Urban	5389 (27.6)	32.6	$$ <0.001$$
Rural	14,172 (72.4)	39.3	
**Source of drinking water**			
Unimproved	4064 (20.8)	43.2	$$ <0.001$$
Improved	15,497 (79.2)	35.9	
**Sanitation**			
Unimproved	7821 (40.0%)	43.1%	$$ <0.001$$
Improved	11,738 (60.0%)	33.7%	

Upon adjusting for socio-demographic characteristics, the results presented in Table 2 suggest a higher odd of childhood diarrhea among children aged 12–23 months (AOR: 2.497; 95% CI: 2.263, 2.755) and 6–11 months (AOR: 2.4; 95% CI: 2.136, 2.695), relative to their counterpart aged 48–59 months. In furtherance, there is a marked incensement in diarrhea risk by approximately 21–51% among children aged 24–35 months (AOR: 1.497; 95% CI: 1.356, 1.652), those ranging from birth to 5 months (AOR: 1.257; 95% CI: 1.112, 1.420), and those between 36 and 47 months (AOR: 1.210; 95% CI: 1.094, 1.338). In terms of gender differences, male children have a higher odd (AOR: 1.142; 95% CI: 1.076, 1.212) of experiencing pediatric diarrhea than female children. Regarding maternal educational attainment, the children of mothers with varying levels of education, from no formal education to upper secondary education, exhibited prevalence odds ratios close to unity as compared to children born to mothers with higher educational levels (No formal education AOR:0.951; 95%CI: 0.808, 1.119, primary education AOR: 1.060; 95%CI: 0.900, 1.248, lower secondary education AOR: 1.112; 95%CI: 0.936, 1.322, upper secondary education AOR: 1.092; 95%CI: 0.925, 1.289), demonstrating no significant association between diarrhea and maternal/caregiver educational level. Furthermore, children born to households classified as poorest and poor encountered increased odds of childhood diarrhea by factors of nearly four (AOR: 3.562; 95%CI: 3.074–4.128) and three (AOR: 3.341; 95% CI: 2.914–3.831), respectively, relative to peers from the richest households. Additionally, significant association between childhood diarrhea and geographic area was noted, with children residing in urban environments exhibiting a one-and-a-half-fold greater likelihood of suffering from childhood diarrhea (AOR: 1.508; 95% CI: 1.363–1.669) compared with their rural counterparts. The study found no statistically significant association between source of drinking water and childhood diarrhea, when comparing unimproved sources (AOR: 0.983; 95% CI: 0.905–1.069). The results further revealed that households relying on unimproved sanitation facilities, such as non-flushing toilets or open latrines, experienced a 21% (AOR: 1.205; 95%CI: 1.121–1.295) increase in the likelihood of childhood diarrhea compared to those with access to improved sanitation systems.

**Table 2:** Factors associated with childhood diarrhea in Yemeni children under the age of five

Characteristic	Unadjusted Prevalence OR	95% $$ CI$$		Adjusted Prevalence OR	95% $$ CI$$	
**Child age (months)**						
0–5	1.279*	1.134	1.443	1.257*	1.112	1.420
6–11	2.302*	2.055	2.578	2.400*	2.136	2.695
12–23	2.422*	2.199	2.667	2.497*	2.263	2.755
24–35	1.478*	1.341	1.629	1.497*	1.356	1.652
36–47	1.201*	1.088	1.327	1.210*	1.094	1.338
48–59	1.00			1.00		
**Gender**						
Male	1.116*	1.053	1.183	1.142*	1.076	1.212
Female	1.00			1.00		
**Mother’s education**						
No formal education	1.489*	1.280	1.730	0.951	0.808	1.119
Primary education	1.497*	1.283	1.748	1.060	0.900	1.248
Lower secondary education	1.370*	1.160	1.617	1.112	0.936	1.322
Upper secondary education	1.340*	1.142	1.573	1.092	0.925	1.289
Higher education	1.00			1.00		
**Household Wealth**						
Poorest	2.605*	2.360	2.875	3.562*	3.074	4.128
Poor	2.423*	2.190	2.682	3.341*	2.914	3.831
Middle	1.915*	1.726	2.124	2.534*	2.219	2.893
Rich	1.728*	1.557	1.917	1.921*	1.722	2.142
Richest	1.00			1.00		
**Place of residence**						
Urban	0.747*	0.699	0.798	1.508*	1.363	1.669
Rural	1.00			1.00		
**Source of drinking water**						
Unimproved	1.357*	1.265	1.456	0.983	0.905	1.069
Improved	1.00			1.00		
**Sanitation**						
Unimproved	1.491*	1.405	1.581	1.205*	1.121	1.295
Improved	1.00			1.00		

* = Significant at $$ p$$-value < 0.05; 1.00 = Reference category

## Discussion

The results of this study reveal that the prevalence of childhood diarrhea among Yemeni children under the age of five is worryingly prevalent at a rate of 37.3%. This prevalence notably surpasses that observed in other Eastern Mediterranean countries facing similar humanitarian challenges attributable to conflict, with Iraq reporting a rate of 12.8% [14], and the State of Palestine, 14.5% [15]. Such statistics paint a troubling picture of the health challenges confronting Yemeni children and indicates the pressing need for targeted interventions to tackle the underlying causes of this health concern.

The analysis further indicates that children in the age group of 6–23 months exhibited a higher odd of diarrhea compared to their younger counterparts aged 0–5 months. This trend aligns with results of previous research conducted on a global scale [16, 17]. A plausible interpretation for this is the protective benefits of exclusive breastfeeding during the initial six months of life, which has been substantiated as a decisive factor in mitigating rotavirus-induced diarrhea [18]. Moreover, post the age of six months, children’s increased mobility through crawling or walking could result in greater exposure to infectious agents [19]. The commencement of complementary feeding, usually past the fifth month mark, also poses heightened risks for diarrhea as it potentially introduces pathogens through contaminated sustenance or water sources [20]. Notably, epidemiological evidence indicates a peak in diarrhea cases during the transition to weaning, when these supplementary feeds are introduced [19, 20].

The results also indicate that the odd of diarrhea is 14% higher in male children compared to female counterpart. This gender disparity in diarrhea is supported by the findings of studies [21, 22], which demonstrated a higher likelihood of diarrhea episodes among male children. This observation, however, contrasts with the research conducted by study [23], which suggested a higher likelihood of diarrhea among female children.

The wealth status of households, as delineated by wealth quintiles, emerged as a pivotal determinant in the prevalence of childhood diarrhea in Yemen. This research revealed a positive gradient in diarrhea risk among children from the poorest wealth quintile, as opposed to children from families in the richest wealth quintile. These findings align with existing research that has identified a notable association between the economic status of a household and the occurrence of diarrhea among children [24–28]. Children from economically disadvantaged households consistently exhibit higher risk of diarrhea compared to their counterparts from the wealthiest households. The influence of households’ wealth on access to healthcare services, and consequentially on health outcomes, has been well established in the literature [29]. Enhancing households’ wealth is imperative for the prevention of diarrhea in developing countries such as Yemen, which necessitates strengthening mother’s livelihoods and combatting poverty [30].

It is noted that there is a discrepancy in the risk of childhood diarrhea among children residing in urban versus rural areas. The unadjusted analysis indicated an increased risk of childhood diarrhea in urban areas, while the adjusted analysis showed the opposite. Our research indicates a higher propensity for diarrhea among children residing in rural localities when compared to their urban counterparts, aligning with findings from previous research [31, 32]. A possible explanation for this discrepancy between urban and rural areas may be the hypothesis that urban environments may lead to increased health problems due to exposure to adverse living conditions [33]. Therefore, this might elucidate the unexpectedly higher rates of diarrhea observed in urban-dwelling children as opposed to those in rural settings.

Moreover, maternal education levels among mothers have been identified as a contributing determinant to childhood illnesses, including diarrhea [34]. The unadjusted prevalence odds ratios indicate a significant association between maternal education and occurrence of diarrhea among children. More precisely, children of mothers or caregivers with limited educational achievements are at greater risk for diarrhea, which in line with the prior research conducted by [35,36, 37]. The link between maternal educational attainment and child health outcomes is intuitive; education equips mothers with critical knowledge to effectively access and utilize health-related information for the benefit of their children’s well-being[38]. The divergence in results was more pronounced among mothers with less than a secondary school education compared to those whose mothers had a higher level of education, although the results also showed some cases of diarrhea in children under the age of five whose mothers were highly educated, indicating that maternal education is not the only determinant of the occurrence of childhood diarrhea and it intersects with other factors. This was reflected by having no significant association between occurrence of childhood diarrhea and mothers/caregivers educational level when adjusting for other sociodemographic and household characteristics. While education in itself may not be completely protective against diarrhea, higher levels of education may be associated with increased health literacy. This can include promoting knowledge of sanitation, hygiene, nutrition and appropriate weaning, which are an integral part of child health [38].

The study findings show an association between unimproved sanitation conditions and the occurrence of diarrhea in children. The results demonstrated that households using inadequate sanitation systems are at an elevated risk of childhood diarrhea relative to households with access to enhanced sanitation facilities. The observed result aligns with results from existing literature, which consistently demonstrate a direct linkage between unimproved sanitation and the incidence of diarrheal (refer to study [39, 40]for an example).

## Conclusion

The findings of this study suggest that age group 6–23, unimproved sanitation, and low-income households are directly related to occurrence of diarrhea in children under five years of age in Yemen.

This study encountered certain limitations. First, owing to the cross-sectional nature of the data used in this study, causal relationships among the variables under consideration cannot be established. Second, the analysis of this study relies on retrospective secondary data, which is inherently prone to issues such as incomplete data. Third, another constraint is the omission of significant factors impacting diarrheal incidence in children and maternal and child health, including access to clean drinking water and domestic water purification practices; these were not factored into the analysis.

Despite these constraints, this study contributes valuable insights into the socio-demographic factors correlated with diarrheal conditions in children aged below five years, derived from an extensive demographic and health survey dataset. Also, the national scope of the MICS data ensures that the insights drawn from this study can be extrapolated to the entire country’s populace. Moreover, this study contributes additional evidence regarding factors that should be prioritized in public health strategies geared towards reducing diarrheal prevalence among children.

## Data Availability

This study used dataset from MICS for Yemen, covering the period 2022 to 2023. This dataset has been made accessible to the public in an anonymized format. Authorization to utilize the MICS Yemen 2022-2023 dataset was contingent upon registration for research purposes through the official MICS website (http://mics.unicef.org/surveys). The MICS program’s website allows for free access to datasets upon request (http://mics.unicef.org/surveys).

## Abbreviations

MICS: Multiple indictor cluster survey
UOR: Unadjusted prevalence odds ratio
AOR: Adjusted prevalence odds ratio
CI: Confidence interval
