# Correction: Statistical Analysis of Factors Associated with Diarrhea in Yemeni Children under Five: Insights from the 2022–2023 Multiple Indicator Cluster Survey

**DOI:** 10.1007/s44197-024-00268-8

**Published:** 2024-06-24

**Authors:** Ali Satty, Mohyaldein Salih, Faroug A. Abdalla, Ashraf F. A. Mahmoud, Elzain A. E. Gumma, Gamal Saad Mohamed Khamis, Ahmed M. A. Adam, Abaker A. Hassaballa, Omer M. A. Hamed, Zakariya M. S. Mohammed

**Affiliations:** 1 Department of Mathematics, College of Science, Northern Border University, Arar, Saudi Arabia; 2 Department of Computer Science, College of Science, Northern Border University, Arar, Saudi Arabia; 3 Department of Finance, College of Business Administration, Northern Border University, Arar, Saudi Arabia

**Journal of Epidemiology and Global Health**

10.1007/s44197-024-00253-1

When this article was published it was not noticed that the five Footnote symbols in the Conclusion section had been accidentally removed.

The Original Article has been corrected.

